# Subcutaneous Emphysema Complicating the Convalescent Stage of Measles in a Malnourished Indigent Child: A Case Report From North-Western Nigeria

**DOI:** 10.7759/cureus.58668

**Published:** 2024-04-21

**Authors:** Aliyu Mamman Na'uzo, Usman A Sanni, Taslim O Lawal, Tawakaltu L Musa, Ojumo O Gabriel, Zainab Abdullahi.K, Muhammad I Habib

**Affiliations:** 1 Paediatrics, Federal Medical Centre, Birnin Kebbi, NGA; 2 Community Medicine, Usmanu Danfodiyo University Teaching Hospital, Sokoto, NGA; 3 Plastic and Reconstructive Surgery, Federal Medical Centre, Birnin Kebbi, NGA

**Keywords:** nigeria, case report, malnutrition, indigent child, subcutaneous emphysema, measles

## Abstract

Measles is a highly infectious, vaccine-preventable viral disease that runs a devastating course in developing countries due to its association with malnutrition and poor immunization coverage. Subcutaneous emphysema (SE) is a rare complication of measles that can be challenging to manage and may portend poor outcomes if untreated.

We present a case of a two-year-old unimmunized rural dweller who presented with facial, neck, and chest swellings three days after being managed for measles exanthem from a referral hospital. Clinical findings were consistent with massive SE comorbid with malnutrition complicating the convalescent stage of measles. The child failed to improve with conservative management but responded to closed thoracostomy tube drainage (CTTD) through an underwater seal bottle with intermittent negative pressure wound therapy (NPWT). The child spent 47 days in the hospital during which the social welfare unit of the hospital supported the treatment.

SE is a rare complication of measles infection that can be challenging to manage, especially when comorbid with malnutrition in an indigent child. The application of a multidisciplinary team approach and the use of CTTD with NPWT may shorten the duration of hospital stay for the patient.

## Introduction

Measles is a highly infectious, vaccine-preventable disease of major public health concern worldwide, especially in developing countries [1]. Despite the availability of a safe and effective vaccine, in 2018 alone, more than 140,000 people, mostly children under the age of five, died from measles [2].

Generally, measles runs a more devastating course in children in developing countries because of the high rate of undernutrition, overcrowding, and lack of access to care, with a mortality rate as high as 1% to 15% [3]. The commonest severe respiratory complication of the disease is pneumonia, which accounts for most measles-related deaths [4]. However, other rare respiratory complications such as mediastinal and subcutaneous emphysema (SE) do occur and are associated with poor outcomes if untreated [5,6].

We present a case of a two-year-old rural dweller with massive SE comorbid with malnutrition following measles. The patient benefited from closed thoracostomy tube drainage (CTTD) through an underwater seal bottle with intermittent negative pressure wound therapy (NPWT) and nutritional support, and a favourable outcome after a prolonged hospital stay.

## Case presentation

The case involves a two-year-old girl who was referred to our Emergency Paediatric Unit from a secondary health care centre with complaints of facial, neck, and chest swellings that developed three days after she was managed and discharged for measles from the referring hospital. She did not receive measles vaccination but had oral polio vaccine (OPV) during the supplementary immunization exercise. She had contact with other children who had measles among members of her household. She was not exclusively breastfed, and her complementary diet was poor. Her current diet was mainly carbohydrates, which she poorly tolerated with the onset of the illness. The parents are of a low socioeconomic class that engages in subsistence farming.

**Figure 1:**
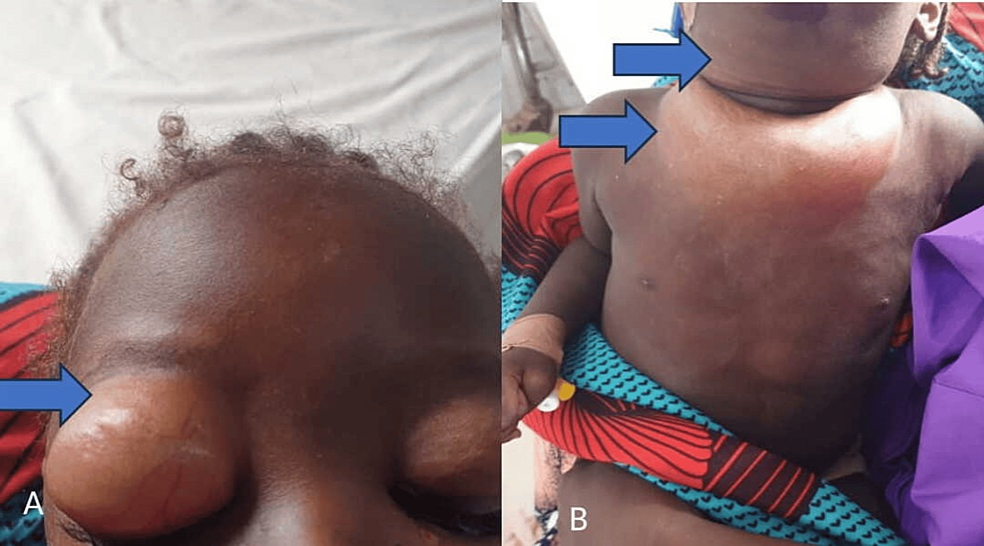
(A) Massive peri-orbital swelling (blue arrow); (B) massive swelling involving the neck, chest, and part of the abdominal wall (blue arrows), crepitus elicited on palpation.

On examination, the patient appeared acutely and chronically ill-looking, wasted and restless, mildly pale with hypo-pigmented hair, angular stomatitis, and desquamating skin lesions on the trunk and upper limbs. She had massive peri-orbital, neck, and chest wall swelling with subcutaneous crepitus extending down to the abdominal wall (Figure 1). She was afebrile with no significant lymphadenopathy. Her mid-arm circumference was 13 cm, her weight was 6.5 kg (54% of the expected), her height was 84 cm, and the Z-score was <-4 SD. Her respiratory system examination showed a rate of 28 cycles per minute with resonant chest percussion and an oxygen saturation (SpO2) of 99%. A provisional diagnosis of convalescent measles complicated by SE on a background of severe acute malnutrition was made.

**Figure 2:**
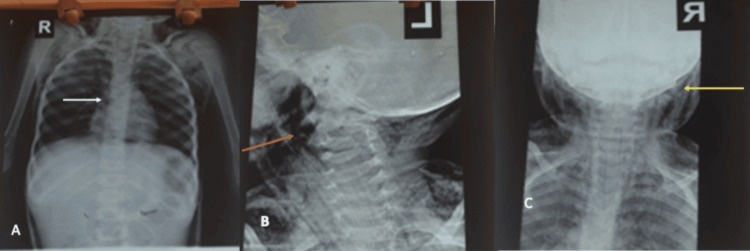
(A) Chest X-ray showing perihilar opacities in the chest. (B, C) X-ray of the neck displaying extensive radiolucency within the subcutaneous tissue (red arrow) and soft tissue swelling (yellow arrow)

Complete blood count showed a WBC count of 10.2 x 10^3^/L with relative leukocytosis, a haematocrit of 28%, and normal platelet counts. Retroviral screening for HIV and reverse transcriptase PCR for COVID-19 were negative. The blood sample for Bact/Alert was positive, and the gram stain showed gram-positive cocci. Sub-culture yielded moderate growth of *Streptococcus* spp. after 48 hours of incubation, sensitive to erythromycin, gentamicin, and linezolid but resistant to vancomycin. An abdominal ultrasound scan revealed a bilateral grade one renal parenchymal disease. Arterial blood gases showed a pH of 7.56, a pCO2 of 51.1 mmHg, a pO2 of 28 mmHg, and an HCO3 of 46.3 mmol/L with a lactate of 2.02 mmol/L. E/U/Cr showed potassium of 2 mmol/L and ionized calcium of 1 mmol/L. Chest X-ray revealed extensive soft tissue swelling in the neck and axillary region with areas of translucency. The lung fields were clear except for the perihilar opacities (Figure 2).

**Figure 3:**
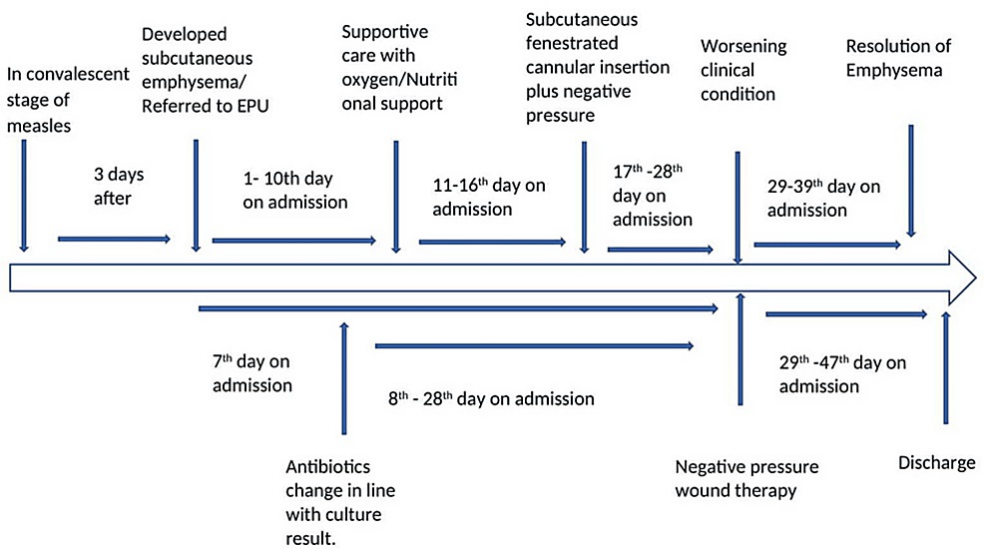
Time course of the patient while on admission

The patient was reviewed by the ophthalmologist, otorhinolaryngologist, paediatric surgeon, and plastic surgeon. She was initially placed on I.V. cefuroxime but changed to I.V. ampicillin/sulbactam and gentamicin, following the blood culture result, which yielded moderate growth of *Streptococcus *spp. after three days of incubation at 37°C. She also had tablets of zinc, vitamin A, and nutritional support with therapeutic milk (F75 and F100). She was transfused with packed cells when the PCV dropped to 20%. Corrections for both potassium and calcium derangements were made with no adverse reactions to either of the medications. Supplemental oxygen was also administered. Her condition initially improved within 48 hours after the change of antibiotic, necessitating the consideration of a gas-producing organism as the cause of the emphysema. However, on the 16th day of admission, her condition worsened. She had subcutaneous fenestrations with an 18G cannula in the supraclavicular area, with the open end attached to a suction machine set at a pressure of 125 mmHg to create negative pressure. She initially improved for five days, but the swelling reaccumulated afterwards. She had closed thoracostomy tube drainage inserted at the right triangle of safety and an underwater seal bottle with intermittent negative pressure drainage on the 28th day of admission with complete resolution of the SE within 10 days. She had a remarkable improvement with healed ulcers, acid-base, and electrolyte balance corrected, gained weight, and was discharged to be followed up in the clinic. Figure 3 shows the time course for the patient while on admission. This article was previously posted to the Research Square preprint server on September 7, 2021.

## Discussion

We report a case of spontaneous SE recalcitrant to treatment in a two-year-old girl in the convalescent stage of measles, comorbid with malnutrition. Reported incidences of this rare complication vary from 0.59% to 1.5% [5]. The usual scenario is that with supportive treatment, the affected children rarely require invasive procedures; the emphysema undergoes resolution within a short period with the eventual recovery of the patient. Air leaks such as mediastinum and SE in patients with measles are postulated to result from the rupture of alveoli due to increased intra-alveolar pressure in the bronchovascular sheet that then travels through fascial planes to the mediastinum and subcutaneous tissues [5,6]. The hypothesis is that increased fragility of connective tissues due to both malnutrition and following the measles infection leads to the formation of bullae and pulmonary interstitial emphysema [5].

The index patient not only had SE complicating a recent attack of measles but also had underlying malnutrition probably accounting for her prolonged hospital stay and recalcitrant course. Although emphysema complicating measles can occur at any age or stage of the disease, in a previous study, only a third of the cases presented during the eruptive stage. Therefore, the possibility of late presentation is of significance in clinical practice since the relationship between the infection and SE may be missed in the absence of a rash, as seen in the present child. In most series, the majority of the cases cluster in children less than five years old, as was the case with the index patient [5,6]. The presentation of massive SE comorbid with malnutrition, in this case, is similar to a case reported by Ahmed et al. from Nguru, north-eastern Nigeria [7]. However, the index patient did not have pneumonia, unlike the child reported by Ahmed et al.

Some differential diagnoses such as trauma, asthma, and tuberculosis, which are common in our environment and can mimic the presentation of the index patient, were ruled out in the course of managing this patient. A study in Italy has reported that the occurrence of SE may be the first sign of tubercular disease in children [8]. Another study, although in an adult patient, has documented the occurrence of pneumomediastinum and SE following SARS-CoV-2 infection [9]. The index patient had both tuberculosis screening and a COVID-19 test, which were all negative. The hilar lymphadenopathies on the chest X-ray may be the residual effect of a previous infection or another pneumonic complication.

The treatment of SE is mainly supportive. However, the index patient could not improve with supportive and minimally invasive management after two weeks of admission. This contrasted with the findings in Kano, Azare, and Somalia [5-7], and necessitated the decision to explore more invasive forms of treatment such as CTTD with NPWT. The outcome of the procedure and the eventual resolution of the symptoms of the child made the parents happy. They also expressed their gratitude for the financial support rendered to them by various stakeholders. A key challenge in managing this patient was the inability to perform a chest CT because of financial constraints. A chest CT would be beneficial in assessing any associated abnormalities such as the presence of a pleuro-cutaneous fistula and in excluding life-threatening differential diagnoses [10].

## Conclusions

We present a case of SE complicating measles infection in an unimmunised child with underlying undernutrition. The management of this child presents key challenges spanning from financial to clinical, ultimately prolonging the hospital stay of the patient. Although the outcome was good, the coexistence of measles and malnutrition might have contributed to the development of complications and prolongation of the hospital stay in this patient. SE is a rare complication of measles and may be a harbinger of a severe form of measles. Further research on this topic will help improve the outcomes of patients.
